# Digital Assessment Tools Using Animation Features to Quantify Alcohol Consumption: Systematic App Store and Literature Review

**DOI:** 10.2196/28927

**Published:** 2022-03-23

**Authors:** Veronika Wiemker, Maria Neufeld, Anna Bunova, Ina Danquah, Carina Ferreira-Borges, Stefan Konigorski, Ankit Rastogi, Charlotte Probst

**Affiliations:** 1 Heidelberg Institute of Global Health Medical Faculty and University Hospital Heidelberg University Heidelberg Germany; 2 WHO European Office for the Prevention and Control of Noncommunicable Diseases Moscow Russian Federation; 3 National Medical Research Center for Therapy and Preventive Medicine of the Ministry of Health of the Russian Federation Moscow Russian Federation; 4 Digital Health Center Hasso Plattner Institute for Digital Engineering University of Potsdam Potsdam Germany; 5 Hasso Plattner Institute for Digital Health at Mount Sinai Icahn School of Medicine at Mount Sinai New York, NY United States; 6 Institute for Mental Health Policy Research Centre for Addiction and Mental Health Toronto, ON Canada; 7 Department of Psychiatry University of Toronto Toronto, ON Canada

**Keywords:** alcohol consumption, harmful and hazardous drinking, screening, assessment methods, eHealth, mobile apps, visualization, animation features, AUDIT, primary health care

## Abstract

**Background:**

Accurate and user-friendly assessment tools for quantifying alcohol consumption are a prerequisite for effective interventions to reduce alcohol-related harm. Digital assessment tools (DATs) that allow the description of consumed alcoholic drinks through animation features may facilitate more accurate reporting than conventional approaches.

**Objective:**

This review aims to identify and characterize freely available DATs in English or Russian that use animation features to support the quantitative assessment of alcohol consumption (alcohol DATs) and determine the extent to which such tools have been scientifically evaluated in terms of feasibility, acceptability, and validity.

**Methods:**

Systematic English and Russian searches were conducted in iOS and Android app stores and via the Google search engine. Information on the background and content of eligible DATs was obtained from app store descriptions, websites, and test completions. A systematic literature review was conducted in Embase, MEDLINE, PsycINFO, and Web of Science to identify English-language studies reporting the feasibility, acceptability, and validity of animation-using alcohol DATs. Where possible, the evaluated DATs were accessed and assessed. Owing to the high heterogeneity of study designs, results were synthesized narratively.

**Results:**

We identified 22 eligible alcohol DATs in English, 3 (14%) of which were also available in Russian. More than 95% (21/22) of tools allowed the choice of a beverage type from a visually displayed selection. In addition, 36% (8/22) of tools enabled the choice of a drinking vessel. Only 9% (2/22) of tools allowed the simulated interactive pouring of a drink. For none of the tools published evaluation studies were identified in the literature review. The systematic literature review identified 5 exploratory studies evaluating the feasibility, acceptability, and validity of 4 animation-using alcohol DATs, 1 (25%) of which was available in the searched app stores. The evaluated tools reached moderate to high scores on user rating scales and showed fair to high convergent validity when compared with established assessment methods.

**Conclusions:**

Animation-using alcohol DATs are available in app stores and on the web. However, they often use nondynamic features and lack scientific background information. Explorative study data suggest that such tools might enable the user-friendly and valid assessment of alcohol consumption and could thus serve as a building block in the reduction of alcohol-attributable health burden worldwide.

**Trial Registration:**

PROSPERO International Prospective Register of Systematic Reviews CRD42020172825; https://www.crd.york.ac.uk/prospero/display_record.php?ID=CRD42020172825

## Introduction

### Background

Alcohol-related injuries and diseases are major causes of morbidity and mortality worldwide, although, at least in theory, they are fully preventable [1]. The well-directed implementation of monitoring, prevention, and treatment programs requires accurate assessment tools to quantify the users’ alcohol consumption. To date, consumption assessments are generally based on standardized self-report questionnaires or brief interviews. At the population level, they form the foundation for public health monitoring, quantification of alcohol-attributable harm, and evaluation of alcohol policies. At the individual level, they constitute the cornerstone of effective harm reduction strategies such as screening and brief intervention (SBI) programs. SBI programs link the routine administration of a screening tool to identify harmful or hazardous drinking, often a questionnaire, to a tailored brief intervention, most commonly comprising a short motivational interview or structured advice [2]. They have been shown to be highly effective in reducing excessive drinking among adults [3,4] and are recommended in national and international policy guidelines for reducing alcohol-attributable harm [5,6]. However, the implementation of SBI programs in public health systems remains low [7,8]. Relevant implementation barriers include a perceived lack of knowledge or skills among health care professionals and environmental context factors such as time restrictions and limited resources [9].

Although the measures used in epidemiological surveys differ between countries and regions [10-12], to date, most of them ultimately require the counting of *standard drinks* consumed. Routine screening tools for primary care such as the Alcohol Use Disorders Identification Test (AUDIT) developed by the World Health Organization [13] and its abbreviated form AUDIT–Consumption (AUDIT-C) [14] also rely on this concept. The standard drink, defined as a beverage volume containing a fixed amount of pure alcohol, facilitates the comparison and assessment of alcohol quantities across different beverage types with varying alcohol content. However, this concept is problematic for 2 main reasons. First, standard drink sizes differ considerably between countries, cultures, and settings, with national definitions varying even within Europe from 8 g of pure alcohol in the United Kingdom to 20 g in Austria [15]. In fact, the majority of countries worldwide do not have an official definition [15]. In addition, relevant AUDIT items are often not adapted to account for differing national standard drink sizes, as required in the AUDIT manual [13,16]. Second, even in countries where the standard drink concept is officially used to standardize the size of retail alcohol, consumers are often not acquainted with the concept and many are unable to convert their consumption correctly [17,18]. For instance, when asked to pour their usual drink and subsequently estimate the number of standard drinks it contained, primary health care patients in the United Kingdom over- or underestimated their actual drink size by at least 0.5 standard drinks in more than half of the cases [19]. In a study conducted among health care professionals in the United States, <20% of the interviewed clinicians could correctly state the volume of a standard drink of liquor [20]. Alongside other known biasing factors, such as memory and social desirability bias or underreporting because of alcohol-related stigma, this might contribute to the considerable underestimation of the total alcohol recorded through official statistics by approximately 50% in nationally representative surveys [21-24].

Evidence suggests that the assessment results of digital and traditional administration modes are comparable in epidemiological surveys as well as in screening situations [25-27]. Promises of digitally administered tools, such as increased standardization and time efficiency, adaptability of the assessment flow based on user input, and seamless integration with electronic health records [28], may thus help address central SBI implementation barriers [29]. Importantly, digital assessment tools (DATs) can replace the standard drink concept by using individualized, interactive animation features to assess the type and amount of alcohol consumed. Related research fields such as nutrition epidemiology have already recognized the usefulness of visualization features to improve the quantification of consumption [30,31].

Currently, there is a growing body of literature focusing on the effectiveness and availability of evidence-based alcohol reduction apps [32,33]. These apps often contain a screening part quantifying the user’s consumption, which might be text based [34] or based on interactive animations [35]. However, to the best of our knowledge, the current availability of interactive animation features in alcohol DATs has not been systematically evaluated. There is also no systematic review of the effects of such features on assessment feasibility, acceptability, and validity.

### Research Questions

This review seeks to answer the following two questions with a focus on DATs quantifying alcohol consumption (alcohol DATs), which use animation to support users in describing their consumption:

1. What freely available animation-using alcohol DATs exist in the English or Russian language, and what are their core characteristics?
2. To what extent have such tools been scientifically evaluated in terms of feasibility, acceptability, and validity?

## Methods

### Study Design

This systematic review was performed in 2 parts. In part 1, an app store search and a web-based search were conducted to identify existing freely available alcohol DATs. Part 2 comprised a systematic literature search to identify studies that evaluated the feasibility, acceptability, and validity of animation-using alcohol DATs.

The study protocol was published in PROSPERO (International Prospective Register of Systematic Reviews; registration number: CRD42020172825) [36]. We adhered to the standards set out in PRISMA (Preferred Reporting Items for Systematic Reviews and Meta-Analyses) 2020 [37]. Where applicable, we also followed the recommendations for methodological reporting of systematic searches in app store environments proposed by Grainger et al [38].

### Part 1: Existing DATs

#### Eligibility Criteria

This review focused on animation-using alcohol DATs, defined as tools that allow the assessment and quantification of the user’s alcohol consumption via an electronic display device (a PC, laptop, or a mobile device). Aiming to include any alcohol DATs using interactive visualizations as opposed to purely text-based quantification tools, a broad definition of the term *animation* was chosen. Specifically, tools were considered to be using animations if they included ≥1 of the following features: (1) selection of a drink or a beverage type from a number of visually displayed options; (2) selection of a drinking vessel from a number of visually displayed glasses and, in some instances, bottles; and (3) simulated interactive *pouring* of a drink—that is, continuously adjusting the beverage level displayed in the chosen drinking vessel. The availability of each of the listed features was recorded to classify the complexity of the animation used. In addition, tools had to allow for the quantification of the user’s alcohol consumption over a defined reference period or occasion, be available in English or Russian, and be accessible free of charge*.* English-language apps form the largest language group among all apps available in the iOS App Store and the Google Play Store [39]. The mentioned app stores represent approximately 95% of the app market share worldwide [40] and offer ≥95% of the available apps free of charge [41,42]. Given the language background of the authors, the review additionally focuses on Russian-language apps. Russia has one of the highest proportions of alcohol-attributable mortality worldwide, and digital health interventions might become part of the promising prevention efforts currently taken and underway [43]. Our search for Russian-language alcohol DATs aims to identify relevant Russian-language alcohol DATs and gauge the potential of repeating the systematic search in additional languages in the future.

#### Search Strategy

The German app store versions of Google Play Store and iOS App Store were searched in June and July 2020, with English as the preferred app language. As app store search functions do not allow the systematic combination of search terms, 4 independent searches were performed on each platform, using the search terms *alcohol*, *alcohol screening*, *alcohol test*, and *drinking*. We recorded the first 250 results per platform using the search term *alcohol*. Given the high overlap between search results and decreasing relevance after the first 50 to 70 results, a maximum of 100 search results were screened for each of the other search terms. The Google search engine was searched in August 2020 using three sets of search terms (*alcohol screening online*, *alcohol test online*, and *drinking test online*). A total of 90 websites were included in the screening.To further explore the extent of regional adaptation in alcohol DATs and potential content differences between national app stores, we conducted additional searches in January and February 2021 in the Russian version of the Google Play and iOS App Store, with Russian as the preferred app language, using translated search terms. All search results were screened. The Russian Google search engine was searched in February 2021.

#### Screening and Selection of Tools

The URLs and titles of all identified app store entries or websites were recorded. After removing duplicates with identical URLs, the remaining app store descriptions and websites were screened for eligibility. A random sample of 25 English app store entries was independently screened by a second reviewer, and agreement was quantified to ensure the objectivity of the eligibility criteria. After screening, potentially eligible mobile tools were downloaded and completed on mobile devices (for English searches: Huawei Honor 9 Lite LLD-L31, Android version 9 and iPhone SE (2016), iOS version 14.0.1; for Russian searches: Samsung Galaxy Tab A 7.0 SM-T285 8 Gb, Android version 9 and Apple iPad (2018), iOS version 11.2). Web-based tools were completed on the web via the Safari and Google Chrome browsers to determine eligibility.

#### Data Extraction

The following data were extracted from the app store entries and linked websites and through testing the apps or web-based tools: general information (tool name, developer, responsible organization, link to website, scientific background or development process, country of publisher, year of the last update, and number of downloads), content features (reference period, underlying questionnaire, feedback on the user’s consumption quantity, use of standard drink concept, target group, and characteristic additional features), and animation features (availability of abovementioned features and options to adjust further drink characteristics).

### Part 2: Studies on Feasibility, Acceptability, and Validity

#### Eligibility Criteria

Part 2 of the review aimed to identify (1) validation studies comparing animation-using alcohol DATs with established assessment methods (eg, paper–pencil, interview, or web-based questionnaires such as the AUDIT [13], AUDIT-C [14], Alcohol, Smoking, and Substance Involvement Screening Test [44], Alcohol Timeline Followback [45]; drinking diaries; standardized clinical interviews; or alcohol biomarkers) and (2) studies reporting on feasibility or acceptability of animation-using alcohol DATs. Eligibility was restricted to completed and fully reported studies. The same eligibility criteria for animation-using alcohol DATs were used as in part 1. When it was not possible to determine whether the eligibility criteria for using animations were met or when a study used a sample of participants aged <15 years, the study was excluded. Studies conducted among general and specialized populations, such as patient populations, were eligible. No geographical, language, or time restrictions were applied.

#### Search Strategy

A systematic literature search was performed using Embase, MEDLINE, PsycINFO*,* and Web of Science. Search terms (Multimedia Appendix 1) were adapted to the requirements of each web-based database with regard to medical subject headings and wildcards. The searches covered publications from January 2000 to August 2020.

#### Screening and Selection of Studies

After removing duplicates, titles and abstracts were screened by a first reviewer, and a subsample of 100 records was independently screened by a second reviewer. In a second step, the full texts were obtained to decide about final inclusion.

#### Data Extraction

Information on general study characteristics (title, authors, year of publication, and type of study), study methods (setting, design, comparator, sample and recruitment strategy, period of data collection, outcomes, and outcome measurement), main findings, and information on the tested alcohol DATs were extracted. Owing to the high heterogeneity of the study designs, no standardized risk of bias assessment was performed.

Where possible, evaluated DATs were accessed and assessed against the same criteria as the DAT identified in the systematic app store search.

## Results

### Part 1: Existing DATs—Tools Identified and Included

#### Overview

**Figure 1:**
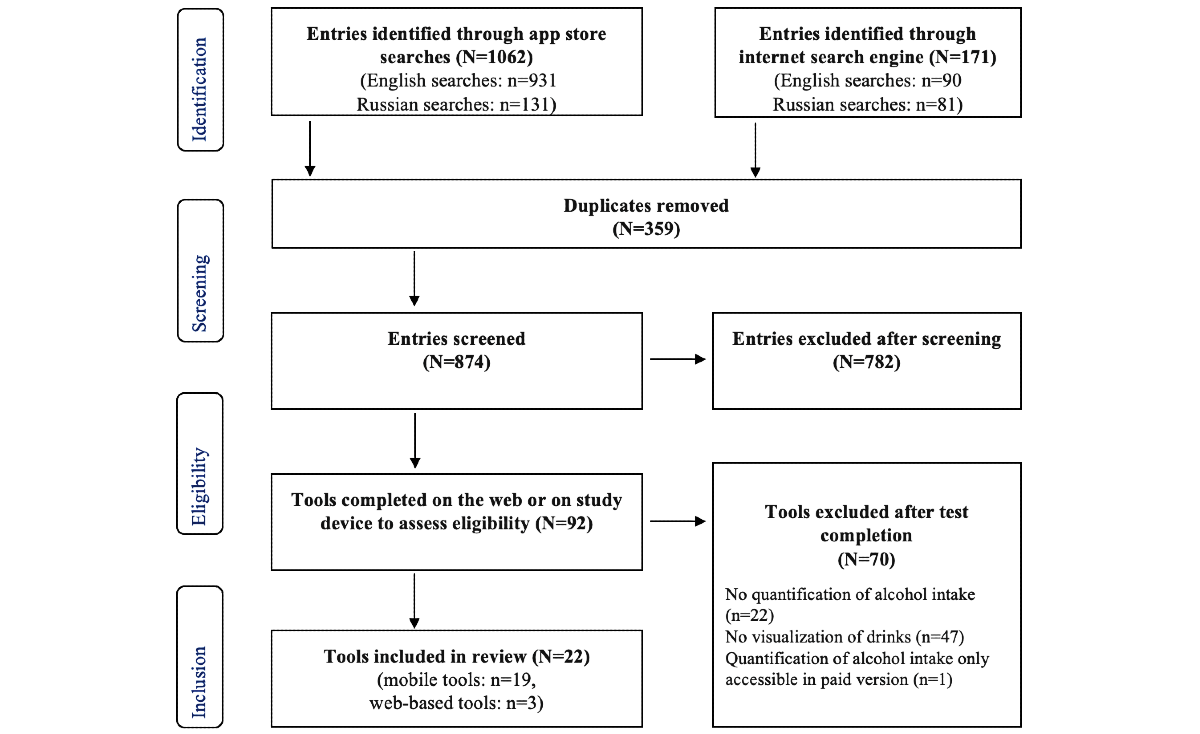
PRISMA (Preferred Reporting Items for Systematic Reviews and Meta-Analyses) flowchart of tool selection.

A total of 1062 app store entries and 171 web entries were identified through app store and web searches (Figure 1). The searches in the Russian language yielded a much lower number of results than the English-language searches. After removing duplicates, of the 1233 total entries, 874 (70.88%) entries were screened for eligible alcohol DATs. Agreement between the reviewers was 92% for exclusion decisions after screening. A total of 54 mobile tools and 38 web-based tools were considered and tested for final inclusion. Finally, 35% (19/54) of mobile tools and 8% (3/38) of web-based tools were eligible. Of the 19 included tools, 16 (84%) were available in English only, 3 (16%) were available in both English and Russian without adaptations in content [46-48], and none were available in Russian only. All included mobile tools were available in German app stores; all but 16% (3/19) of mobile tools [49-51] could also be downloaded from Russian app stores.

#### Content Characteristics

The core characteristics of the 22 alcohol DATs are summarized in Table 1 (detailed in Multimedia Appendix 2) [46-65]. Of the 19 mobile tools, 9 (47%) were available for both iOS and Android operating systems, 5 (26%) were published only in the iOS app store, and 5 (26%) were published only in the Google Play Store. The year of the last update ranged from 2014 to 2020, with 55% (12/22) of tools updated in 2020 or the previous year. The download numbers (only available for Android apps) ranged from ≥100 [49] to 50,000 [48,52]. Approximately 36% (8/22) of tools were developed in the United Kingdom [51-57], and 5% (1/22) each in Ireland [49], Canada [58], Russia [47], France [48], Denmark [46], Germany [59], and Japan [50]. The country of origin could not be identified for 18% (4/22) of tools [60-62,66]. There was no information available regarding any regional or cultural adaptation, and for tools available in both Russian and English, no cultural adaptations were evident. Publishing institutions included public actors [51,53,58], registered charities in the field of alcohol use prevention and general health [52,56,63], and private companies [46-48,54,55,59]. No information about the legal status of the publishing institution could be identified for 32% (7/22) of tools [49,50,57,61,62,66]. For only 14% (3/22) of tools, a scientific background and development process was mentioned [51,58,59]. With the exception of 5% (1/22) of tools designed for health care professionals [51], all tools targeted the general adult population, with a focus on individuals wanting to monitor or cut down their alcohol consumption.

Out of 22 identified tools, 3 (14%) were primarily designed to *assess risky drinking in a one-time screening* [51,53,54] and led to a structured feedback section, including or enabling (1) an estimate of the user’s alcohol-related health risk, (2) a comparison of the individual consumption to a relevant guideline or reference group, and (3) additional information on the standard drink concept and alcohol-related health risks. A total of 14% (3/22) of the identified tools were designed to deliver *individualized programs to reduce or quit drinking* [46,58,59] and started with a brief prospective [59] or retrospective [46,58] assessment of the user’s baseline consumption, followed by a tailored reduction scheme. All remaining tools relied on real-time assessment and were designed to either estimate the users’ blood alcohol concentration (*blood alcohol concentration calculators*; 4/22, 18% of tools) [50,60,63,66], count the number of standard drinks at a drinking occasion (*standard drink counters*; 1/22, 5% of tools) [48], or keep track of the alcohol consumed over a longer period (*drinking diaries*; 8/22, 36% of tools) [47,49,52,56,57,61,62]‬‬‬‬‬‬‬‬‬‬‬‬‬‬‬.

Although none of the tools relied on the standard drink concept in the assessment part, most (13/22, 59% of tools) referred to this concept in their results and feedback sections [48,51-58,61,63,66,67]. In addition to alcohol-related health risk and consumed alcohol quantity, 45% (10/22) of tools reported money spent on alcohol, calories consumed, and hypothetical money or calories saved by cutting down drinking [48,50,52-56,58,61,63]‬‬‬‬‬‬‬‬‬‬‬‬‬‬‬‬‬.

Similar to the mobile tools, all 3 included web-based alcohol DATs addressed the general adult population. They were provided by nonprofit organizations from Ireland [64,65] and the United Kingdom [56], with copyright claims absent [56] or dating to the current (2020) [65] or past year [64]. All organizations provided contact details of support services helping to cut down drinking. Out of 22 identified tools, 2 (9%) [56,64] were digital versions of the World Health Organization’s AUDIT. The functionality of standard drink calculation was directly embedded into AUDIT-C item 3 (“How many units of alcohol do you drink on a typical day when you are drinking?”), preserving the questionnaire’s original item structure. Both led to a detailed feedback section, including AUDIT score and risk category, information on standard drinks, and calories consumed on a typical day. The third tool converted the user’s reported consumption into standard drinks [65] and provided additional feedback items, including a comparison of the user’s alcohol consumption to a weekly low-risk drinking guideline [68].‬‬‬‬

**Table 1:** DATs^a^ quantifying alcohol consumption (alcohol DATs) in the English language: core characteristics of the included tools (N=22).

Tool name (year of last update^b^; country)		Animation features				Adjust drinks^c^ (n=13)		User feedback			Extra features
		*Drinks*^d^ (n=21)	*Vessels*^e^ (n=9)	*Pour*^f^ (n=2)			Unit of consumption^g^		Additional feedback^h^		
**Mobile app: 1-time assessment of risky drinking**											
	Drinks Meter (2020; United Kingdom) [54]	✓	✓		✓		Standard drinks		Physiology or nutrition	Text-based AUDIT^i^; *drink pourer* tool	
	Know Your Numbers (2017; United Kingdom) [51]	✓	✓				Standard drinks		—^j^	Alcohol unit guide	
	Know Your Units (2017; United Kingdom) [53]	✓			✓		Standard drinks		Physiology or nutrition	Beverage-specific sound animations	
**Mobile app: individualized program to reduce or quit drinking**											
	MeSelfControl (2016; Germany) [59]	✓	✓		✓		Alcohol quantity		—	—	
	ReduceYour Drinking (2015; Denmark) [46]	✓					Alcohol quantity		—	Text-based DATs; available in Russian	
	Saying When (2016; Canada) [58]	✓	✓	✓			Standard drinks		Positive effect	Explanation of standard drink concept	
**Mobile app: BAC** ^k^ **calculator**											
	alcCalc (2014: Japan) [50]	✓					Alcohol quantity		Physiology or nutrition	—	
	Alcohol Diary (2019; not provided) [67]	✓					Standard drinks		—	—	
	Alcohol meter (2019; not provided) [60]			✓	✓		Alcohol quantity		Physiology or nutrition	—	
	DrinkWatch Unit Checker (2016; United Kingdom) [63]‬‬‬‬‬‬‬‬‬‬‬‬‬‬‬	✓		✓	✓		Standard drinks		Physiology or nutrition; negative effect	—	
**Mobile app: drinking diary**											
	AlcoExpert (2019; Russia) [47]	✓			✓		Alcohol quantity		Physiology or nutrition; negative effect	Photorealistic drink images; available in Russian	
	Alcofy (2020; not provided) [62]	✓	✓		✓		Alcohol quantity		Physiology or nutrition	—	
	DrinkCoach (2020; United Kingdom) [56]	✓			✓		Standard drinks		Physiology or nutrition; positive effect	Visualized drinking scene; link to animation-enhanced AUDIT	
	DrinkControl (2020; not provided) [61]	✓			✓		Standard drinks		Negative effect	Photorealistic drink images	
	Dry Days (2020; United Kingdom) [55]	✓	✓		✓		Standard drinks		Positive effect	—	
	Drynk (2020; Ireland) [49]	✓			✓		Standard drinks		—	BAC calculator	
	Simple Alcohol Unit Tracker (2020; United Kingdom) [57]	✓					Standard drinks		Negative effect	—	
	Try Dry (2020; United Kingdom) [52]	✓	✓		✓		Standard drinks		Physiology or nutrition; positive effect	AUDIT-C^l^	
**Mobile app: SD counter**											
	Wise Drinking (2019; France) [48]	✓	✓		✓		Standard drinks		Physiology or nutrition	Available in Russian	
**Web-based tool: 1-time assessment of risky drinking**											
	DrinkCoach Alcohol Test (not provided; United Kingdom) [56]	✓	✓				Standard drinks; AUDIT risk score		Physiology or nutrition	Visually enhanced AUDIT; linked to the DrinkCoach mobile tool	
	HSE Self-assessment tool (2019; Ireland) [64]	✓					Standard drinks; AUDIT risk score		Physiology or nutrition	Visually enhanced AUDIT	
**Web-based tool: SD counter**											
	Drinkaware Drinks Calculator (2020; Ireland) [65]	✓					Standard drinks		Physiology or nutrition; negative effect	Drink selection depends on chosen drinking context	

^a^DAT: digital assessment tool.

^b^At time of data extraction (2020).

^c^Nonvisually adjust drink characteristics.

^d^Choose drinks from visual selection.

^e^Choose vessels from visual selection.

^f^Simulated interactive *pouring* of drinks.

^g^Standard drinks, alcohol quantity (pure ethanol consumed [eg, in g or L]), and AUDIT risk score.

^h^Physiology- or nutrition-related feedback (eg, calories, ingested sugar, alcohol quantity equivalent in volume of beer or vodka, *burger equivalent*, exercise time to burn calories, typical symptoms at intoxication level, time until sober); negative effect of consumption (eg, money spent, heavy drinking days, drinking days per week); positive effect of reduced consumption (eg, money saved, sober days).

^i^AUDIT: Alcohol Use Disorders Identification Test.

^j^Not available.

^k^BAC: blood alcohol concentration.

^l^AUDIT-C: Alcohol Use Disorders Identification Test–Consumption.

#### Use of Animation

The 3 animation features defined in the eligibility criteria represent different levels of animation complexity. The distribution of these animation features in the identified alcohol DATs is summarized in Textbox 1.

**Figure 2:**
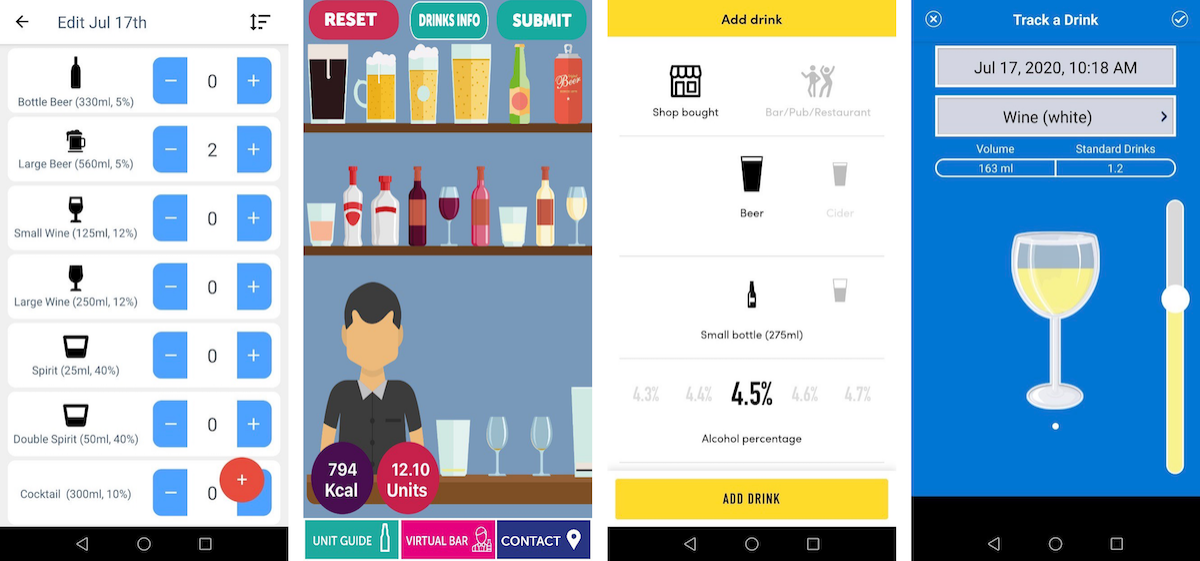
Screenshots of drink input sections in mobile digital assessment tools quantifying alcohol consumption. From left to right: Simple Alcohol Unit Tracker [58] and Know Your Units [54] (both 1-step visual description); Try Dry [53] (2-step visual description); Saying When [59] (2-step visual description with adjustment of the vessel fill height).

With the exception of 9% (2/22) of tools using photorealistic images [47,61], all tools presented a selection of abstract and often simplified drink icons. Examples of the assessment screens are shown in Figure 2. More than half of the tools (12/22, 55%) offered only 1 animation feature (*selection of a drink or a beverage type from a number of visually displayed options*). The number of drinks to choose from differed considerably. Tools with fewer choices (<10 drink icons) [46,49,50,57,63,64,66] did not allow for any individualization of the chosen drink, whereas tools with more choices (16-29 drink icons) [47,56,61] enabled the user to individually adjust certain drink characteristics, including standard units of alcohol [56], drinking vessel size [61], volume consumed [47], and alcohol content of the consumed beverage. The *Know Your Units* tool [53] featured a *virtual bar* animation [53], allowing the user to drag a predefined drink icon from a shelf onto a bar table, where it was emptied out, accompanied by a beverage-specific sound animation.

Most other tools (8/22, 36%) relied on a 2-step process to describe consumed drinks. After choosing a beverage category, users could choose their glass or bottle from a beverage-specific selection. In the group of mobile tools, the choice of available beverage categories and vessels per category differed from basic (3-7 beverage categories; ≤3 vessel icons per category) [48,59,62] to detailed (6-7 beverage categories; 4-10 vessel icons per category) [52-55]. All but 25% (2/8) of these tools [48,59] displayed all individualization steps on 1 overview screen (eg, Figure 2, *Try Dry*). All tools allowed for nonvisual adjustment of beverage alcohol content [48,52-55,59] or consumed beverage quantity [59,62]. The *DrinkCoach* web-based tool [56] lets users choose from 12 beverage categories, as well as 3 to 7 vessels per beverage category, but did not allow for further adjustment of drink characteristics.

Only 9% (2/22) of the included tools featured the simulated interactive *pouring* of a drink; that is, continuously adjusting the beverage level displayed in a drinking vessel [58,60]. One of these tools used a nonchangeable standard vessel icon and a standard-colored beverage for the animation [60]. The other tool allowed users to choose the beverage and the vessel before pouring their drink (Figure 2, *Saying When*) [58]. The poured volume was displayed in real time during the pouring action, in milliliters as well as in standard drinks. The color of the liquid matched that of the chosen beverage. Further features to enhance the 3D character of the pouring experience, such as shadows, sound animations, or pouring-induced movement of the liquid surface were not identified.

**Identified animation features in mobile and web-based alcohol digital assessment tools.**

**Identified animation features**

- Most of the included tools (21/22, 95%) offered the selection of a drink or a beverage type from a number of visually displayed options (1-step visual description).
- Less than half of the tools (9/22, 41%) additionally offered the selection of a drinking vessel from a number of visually displayed glasses and, in some instances, bottles (2-step visual description).
- Only 9% (2/22) of tools allowed the simulated interactive pouring of a drink; that is, continuously adjusting the beverage level displayed in the chosen drinking vessel.

### Part 2: Identification of Feasibility, Acceptability, and Validity Studies

#### Overview

**Figure 3:**
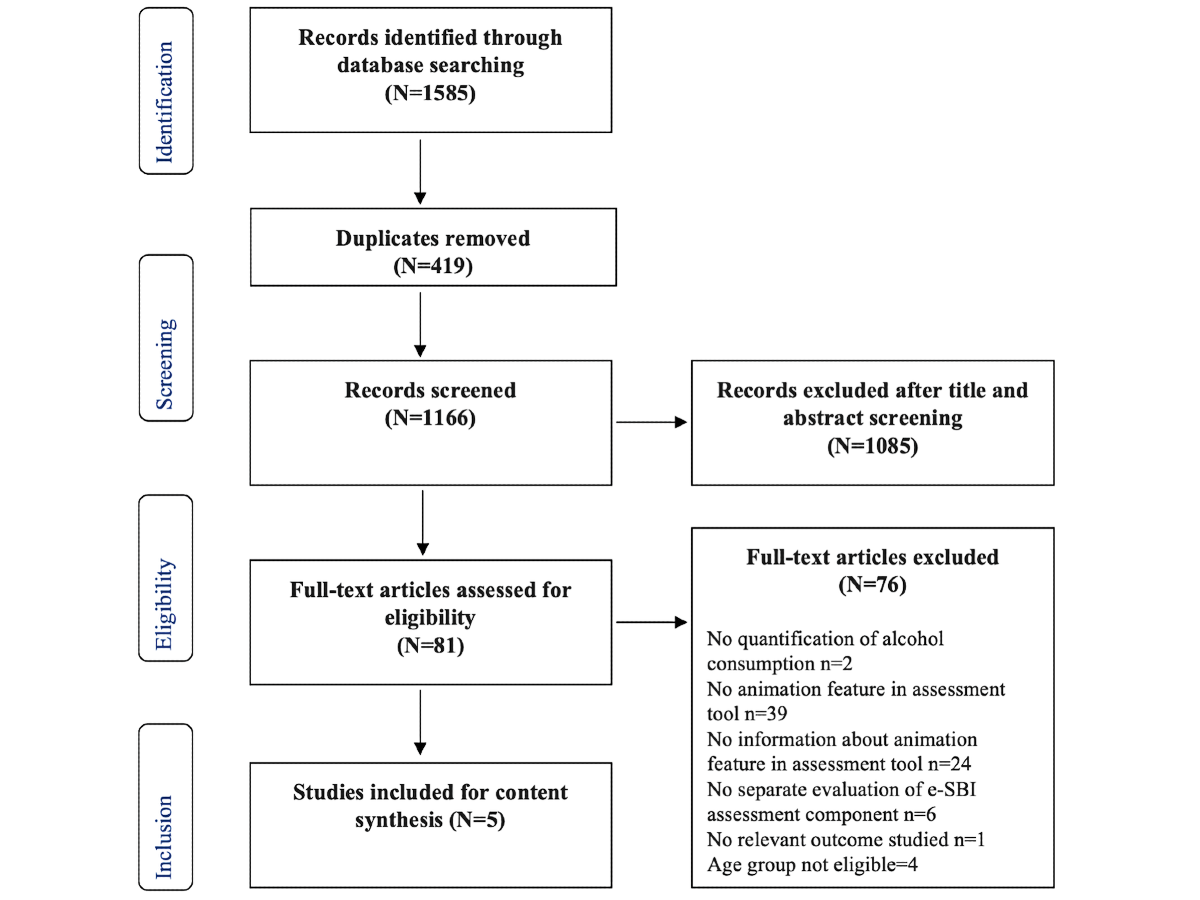
PRISMA (Preferred Reporting Items for Systematic Reviews and Meta-Analyses) flowchart of study selection. eSBI: electronic screening and brief intervention.

A total of 1585 records were identified through a systematic literature review search in Embase, MEDLINE, PsycINFO, and Web of Science (Figure 3). Removal of duplicates left 73.56% (1166/1585) of records for the title and abstract screening. In the random sample of 100 records screened by 2 reviewers, the agreement was 92% for inclusion decisions. Of the 81 full-text articles assessed for eligibility, 5 (6%) met the inclusion criteria.

#### Study Characteristics

All included studies used a 1-arm study design with convenience sampling to explore the feasibility, acceptability, or validity of the alcohol DAT in question, or several of these concepts (Table 2). Of the 5 studies, 3 (60%) were conducted in Australia [69-71], 1 (20%) in Canada [72], and 1 (20%) study used a combined sample of participants recruited in Canada and Switzerland [73]. Data collection took place between 2015 and 2017; 40% (2/5) of papers [70,71] did not report the period of data collection.

Participants were recruited at primary health care and addiction centers [69,71] and through researcher networks [70], advertisements on university campus [70,72], social media, and internet forums [70,73]. Of 5 studies, 2 (40%) recruited participants from the general population [70,72]; 20% (n=1) of studies focused on adults with risky alcohol use [73]; and 40% (n=2) of studies used quotas to include nondrinkers, nondependent drinkers, and dependent drinkers [69,71]. Sample sizes ranged from 50 [72] to 671 [70] participants, with balanced proportions of men and women.

**Table 2:** Overview of included studies (N=5).

Reference			Country; time of data collection (tested alcohol DAT^a^)		Study sample (age in years)		Recruitment		Main findings on acceptability and criterion or convergent validity
**Acceptability and feasibility studies**									
	Lee et al [69]	Australia; 2016-2017 (Grog Survey app)		246 patients (18-78) with and without problematic alcohol use; 5 field research assistants (—^b^)		Primary health care and addiction center		Acceptability: 97% of patients rated alcohol DAT as easy to use or okay to use (rather than hard to use); staff suggested a high potential for the app to be used in primary health care settings, noted that participants appeared engaged and required minimal assistance	
	Bertholet et al [73]	Switzerland and Canada; 2015 (Alcooquizz)		130 participants (mean 32.8, SD 10) with problematic alcohol use		Social media and internet forums		Acceptability: Low self-reported frequency of alcohol DATs use during the 3-month study period (only 53.6% of participants reported using it more than once); moderate rating for appreciation and usefulness of the alcohol DAT (mean 6/10 points, IQR 5-8)	
**Validation studies**									
	Lee et al [71]	Australia; 2019^c^ (Grog Survey app)		238 participants (18-78) with and without problematic alcohol use		Primary health care and addiction centers		Criterion and convergent validity: Moderate (Spearman correlation between alcohol DAT and clinical interview for consumption quantity: r=0.68; *P*<.01); compared with interviews, alcohol DAT recorded higher numbers of standard drinks consumed per drinking occasion (median 17.0, IQR 10.5-27.9 and median 15.4, IQR 9.6-23.2) Criterion validity: Equal or better correlation of the presence of self-reported withdrawal tremors with the self-reported quantity of alcohol consumption in the alcohol DAT (r=0.40; *P*<.05) than with consumption estimate in the clinical interview (r=0.32; *P*<.05)	
	Poulton et al [70]	Australia; 2018^c^ (CNLab-A)		671 participants (16-56) with unknown alcohol use		Researcher networks and social media and internet forums		Convergent validity: Acceptable or high, with a significantly higher percentage of drinking days (*P*=.007) and total alcohol intake (*P*<.001) assessed by EMA^d^ alcohol DAT compared with 21-day TLFB^e^; alcohol DAT recorded significantly higher hourly alcohol intake compared with AUQ^f^ (*P*=.002); no significant difference between AUQ and DAT in estimated weekly average consumption (*P*=.13)	
	Vanderlee et al [72]	Canada; 2016 (Beverage Frequency Questionnaire)		50 participants (16-30) with unknown alcohol use		Advertisement on university campus		Convergent validity: High correlation with 7dFR^g^ for number of drinks (Pearson r=0.58; *P*<.001) and consumed volume (r=0.78; *P*<.001) Acceptability: Good comprehensiveness assessed through cognitive interviewing (78% of participants reported no trouble in selecting a beverage image).	

^a^DAT: digital assessment tool.

^b^Not available.

^c^Year of study, as the year of data collection is not available.

^d^EMA: ecological momentary assessment.

^e^TLFB: Alcohol Timeline Followback.

^f^AUQ: Alcohol Use Questionnaire.

^g^7dFR: 7-day food record.

#### Characteristics of the Evaluated Tools

The evaluated alcohol DATs included 3 mobile apps and 1 web-based tool (Table 3), none of which had been identified in this review through the systematic app store and web search. Only 25% (1/4) of tools were publicly available in the German or Russian app stores [73]. They were designed to screen for risky alcohol use and collect consumption data at the population level [69,71], enable real-time assessment of alcohol intake [70], deliver a program to reduce drinking [73], and conduct epidemiological research [72].

Of the 4 tools, 2 (50%) presented a low number of visually displayed drink choices (<10 drink icons) [70,73]; 25% (1/4) of tools offered the additional choice of a drinking vessel (16 vessel icons in 4 alcoholic beverage categories) [72]. The *Grog Survey app* offered a wide range of region- and culture-specific beverages and drinking vessels and the additional feature of *pouring* a drink [71,75].

**Table 3:** Scientifically evaluated DATs^a^ quantifying alcohol consumption: overview of core characteristics.

Tool name (year of study)		Animation features			Adjust drinks^b^	User feedback			Extra features
		*Drinks^c^*	*Vessels^d^*	*Pour^e^*		Unit of consumption^f^	Additional feedback^g^		
**eSBI^h^for problematic alcohol use (mobile app)**									
	Alcooquizz (2017) [73]	✓				Risk score	Physiology or nutrition; negative effect	Comparison to reference group	
**Ecological momentary assessment alcohol DAT (mobile app)**									
	CNLab-A (2018) [70]	✓			✓	N/A^i^	—^j^	—	
**One-time assessment of risky drinking (mobile app)**									
	Grog Survey app (2019) [71,74]	✓	✓	✓		AUDIT^k^ risk score	—	Visualizations partly use user-generated drinks	
**One-time alcohol consumption assessment for epidemiological research (web-based)**									
	Beverage Frequency Questionnaire (2018) [72]	✓	✓			N/A	—	Also assesses consumption of nonalcoholic drinks	

^a^DAT: digital assessment tool.

^b^Nonvisually adjust drink characteristics.

^c^Choose drinks from visual selection.

^d^Choose vessels from visual selection.

^e^Simulated interactive *pouring* of drinks.

^f^Standard drinks, alcohol quantity (pure ethanol consumed [eg, in g or L]), AUDIT risk score, and DAT designed for epidemiological research, did not report the results to the user.

^g^Physiology or nutrition-related feedback (eg, calories, ingested sugar, and alcohol quantity equivalent in volume of beer or vodka, *burger equivalent*, exercise time to burn calories, typical symptoms at intoxication level, and time until sober); negative effect of consumption (eg, money spent, heavy drinking days, and drinking days per week); positive effect of reduced consumption (eg, money saved; sober days)

^h^eSBI: electronic screening and brief intervention.

^i^N/A: not applicable; DAT designed for epidemiological research, did not report the results to the user.

^j^Not available.

^k^AUDIT: Alcohol Use Disorders Identification Test.

#### Findings Regarding Acceptability and Feasibility

Of the identified 5 studies, 2 (40%) focused on the acceptability and feasibility of the evaluated alcohol DAT (Table 2) [69,73]. Both used participant rating scales, rating *appreciation* and *usefulness* [73] and *ease of use*, respectively [69]. One of the tools, which was offered to study participants to be used at their discretion during a 3-month period, recorded the self-reported frequency of use [73]. In the second study, conducted in a health care setting, quantitative and qualitative staff observations were taken into account.

User evaluations of alcohol DATs were moderate to favorable. The animation-using personal feedback module of the first tool received an average participant rating of 6/10 in both the *appreciation* and the *usefulness* scales [73]. However, the self-reported frequency of use was low. The second tool was rated as *easy to use* or *okay to use* rather than *hard to use* by 97% of the study participants. Staff observations concluded that it could be completed with or without minimal assistance across different age groups [69].

#### Findings Regarding Validity

In total, 60% (3/5) of studies aimed to explore the validity of the respective alcohol DAT [70-72]. Established assessment methods to quantify alcohol consumption, such as clinical interviews or the alcohol Timeline Followback questionnaire, were used as comparators. One of the studies additionally evaluated the correlation between physical signs of addiction and the self-reported quantity of alcohol consumption [71].

The reported convergent validity was moderate in one of the studies [71] and moderate to high in a second study [72]. In 40% (2/5) of studies, the alcohol DAT recorded higher alcohol consumption than the established assessment method [70,72]. In one case, comparing an alcohol DAT designed for real-time drinking assessment with a 21-day retrospective assessment, this difference was statistically significant for the percentage of drinking days and the total alcohol intake but not for the number of heavy drinking occasions [70]. In the other study, the number of standard drinks consumed per drinking occasion did not significantly differ between the alcohol DAT and the established assessment method [71]. Furthermore, consumption estimates recorded in the alcohol DAT predicted physical signs of addiction as good or better than a clinical interview [71].

## Discussion

### Principal Findings

This systematic review is the first on DATs using animation features to support the quantitative assessment of alcohol consumption, a novel approach in the emerging field of digital health. Only 9% (2/22) of the alcohol DATs identified in part 1 of the review used animation in the sense of dynamically animated images that can be modified through user interaction (*pouring* a drink). Most animation features were implemented in a simplistic manner and did not exploit the full visualization potential of the available technology. The addition of dynamic visual hints, such as foam, bubbles, or visible movement of the beverage, could potentially help users recall their drinking habits in greater detail, which is thought to enhance the accuracy of reporting [76,77]. The results indicate that these features remain underused and that there is ample room for exploration and development.

In the identified alcohol DATs, relevant information regarding the responsible organization, scientific background, and development process was often incomplete or unavailable, which prevented a well-founded quality assessment. A larger degree of transparency is urgently required to fully exploit the potential of animation-using alcohol DATs. Similarly, none of the included tools provided information on the cultural or regional adaptation of the offered beverages and drinking vessels [15,78] or the approach and data sources used for this process. Additional searches with Russian search terms, aiming to identify relevant Russian-language alcohol DAT and gauge the potential of repeating the systematic search in additional languages in the future, showed a high availability of English-language apps in Russian app stores. However, they did not yield evidence of efforts to account for different cultural contexts in different language versions of the same app*.* Moreover, to the best of our knowledge, none of the tools identified in the first part of the review had been scientifically evaluated, underlining the lack of evidence for animation-using alcohol DATs publicly available in app stores [79].

The second part of this review identified 5 exploratory studies on the feasibility, acceptability, and validity of 4 animation-using alcohol DATs. These data showed fair to high convergent validity between established consumption assessment methods and animation-using alcohol DATs, whereas some alcohol DATs were shown to record higher quantities of alcohol consumption than the established measure. Considering the worldwide underestimation of self-reported alcohol consumption [22-24], these results could arguably be interpreted as a sign of improved assessment accuracy [21]. Animation-using alcohol DATs might thus contribute to reducing the well-known bias of standard surveys.

### Strengths and Limitations

To not miss any relevant alcohol DAT using interactive visualizations, as opposed to purely text-based quantitation tools, a broad definition of the term *animation* was chosen, encompassing any apps that allow an image-based interaction with the user to quantify personal alcohol consumption. In part 1, systematic searches were conducted in Android and iOS app stores and via the Google search engine. These sources do not provide access to tools that are published in smaller stores, such as Amazon App Store, Samsung Apps, or Windows Store, or on open-source platforms, such as Github [80]. Alcohol DATs developed for health institutions or researchers may also have been missed, as they often use ways of dissemination not covered in this review [81]. We restricted our search to apps available free of charge. More than 95% of the apps in the Android market [41] and >99% of all downloaded and installed apps [42] are estimated to meet this criterion.

The specific limitations of part 1 stem from the characteristics of nonscientific search engines and app stores as search environments. The providers of the platforms searched for this review do not disclose their search algorithms [38,82]. Search parameters, such as language and region settings and customization based on previous search behavior, are known to influence the choice and order of results, reducing the replicability of searches. Copyright regulations and the differing contents of national app stores further limit the selection of apps available for review. Separate searches in all available national app stores would not have been feasible with the available resources. On the basis of the team’s locations, we searched the German and Russian app stores, which showed a high content overlap with US and UK app stores [39]. The searches allowed for the identification of alcohol DATs from several countries. Obtaining true global or regional representativeness is beyond the scope of this review.

Moreover, digital app stores can be considered very unstable sources of information. Their contents change quickly over time and although for research articles stable identifiers, such as the digital object identifier number, have been developed, so far, there is no equivalent for mobile apps. Analyzing app store data through systematic searches is a relatively novel approach; thus, accepted reporting guidelines are not yet available. However, first recommendations have been developed [38,82], which guided the reporting in this review.

Many studies identified in part 2 focused on the evaluation of electronic SBI programs to reduce alcohol consumption and provided no information on the use of animation features in the alcohol DAT that was tested. If this information could be obtained through a web search, the study was excluded from the review. Therefore, it is possible that some studies evaluating animation-using alcohol DATs were falsely excluded.

### Comparison With Prior Work and Future Research Perspectives

Today, >318,000 health apps are available on the app stores [83], most of which are not recognizably evidence based [79,84]. Many apps, especially in the field of alcohol use, even promote harmful behavior [85]. Efforts have been undertaken to develop frameworks for app quality evaluation [86,87], as well as provide systematic evaluations of the apps available in specified health fields [88-90]. There are promising data on both the efficacy of health apps to reduce harmful alcohol consumption [32,33] and on the effect of interactive elements and gamification in health apps [91-93]. This review adds a separate evaluation of the availability and effects of interactive animation features on alcohol DAT.

Further research is needed to evaluate the differences between regional app markets within and beyond the English- and Russian-language markets. To facilitate the implementation of animation-using alcohol DAT in existing health care systems, target group–specific evaluations, analyzing the perspective of different age groups, and professional versus patient experiences with animation-using alcohol DAT would be highly valuable. The cognitive and psychological mechanisms underlying the effects of animation features also warrant further evaluation. For future research and tool development, the field of alcohol assessment might benefit from deepening the dialog with nutritional and dietary studies that have already started the development of interactive tools using more elaborate animation features that showed high validity and user-friendliness [30,94,95].

### Conclusions

Research in the field of DATs is rapidly advancing. This is especially true for the area of mental health assessment tools, platforms, and resources and seems particularly urgent in light of the current COVID-19 pandemic [96-98]. By facilitating the collection of internationally comparable data as part of population-based surveys and improving the delivery of electronic SBIs for hazardous and harmful alcohol use, animation-using alcohol DATs might contribute to reducing alcohol-attributable health burden in the future. However, the potential of using animation features for the quantification of individual alcohol intake in DATs has not been fully exploited to date and has received little scientific attention. Further research is needed to explore the extent to which such features could improve the accuracy and user-friendliness of the assessment and identify the underlying mechanisms. However, although mostly using nondynamic animation features and often deficient in scientific background information, first animation-using alcohol DATs are available in app stores and on the web, and the explorative study data generated so far support their novel approach.

## Appendix

Multimedia Appendix 1 Keyword set (Ovid search).

Multimedia Appendix 2 Included digital assessment tools.

## Abbreviations

AUDIT: Alcohol Use Disorders Identification Test
AUDIT-C: Alcohol Use Disorders Identification Test–Consumption
DAT: digital assessment tool
PRISMA: Preferred Reporting Items for Systematic Reviews and Meta-Analyses
PROSPERO: International Prospective Register of Systematic Reviews
SBI: screening and brief intervention
