# Supplementary material for: Digital Assessment Tools Using Animation Features to Quantify Alcohol Consumption: Systematic App Store and Literature Review
Source: J Med Internet Res. 2022 Mar 23;24(3):e28927. doi: 10.2196/28927 (PMC8987963; doi:10.2196/28927)
Supplement: Multimedia Appendix 1 [file jmir_v24i3e28927_app1.doc]

## Appendix 1 - Keyword set (Ovid search)

| 1: Alcohol use | (alcohol *abuse/ OR (alcohol abuse).ti,ab.  OR ("alcohol use disorder").ti,ab.  OR (alcohol addiction).ti,ab.  OR (alcohol dependence).ti,ab.  OR exp *Alcoholism/di, ep  OR exp *Drinking Behavior/ OR exp *Alcohol Drinking/ OR exp *Drinking/ OR *Binge Drinking/ OR (alcoholic beverages).ti,ab. OR (alcohol and (drinking or intake or consumption)).ti,ab.) |
| --- | --- |
| 2: Electronic device/digital application  OR  Tool included in review | exp *Mobile Applications/ or (Smartphone or "Mobile application" or Tablet or "web?site" or website or online or m$health or e$health or "browser?based" or browser-based).ti,ab. or Electronic.ti,ab or App.ti,ab. or exp *Software/ or exp *Cell Phone/  OR (alcCalc or "AlcoExpert: Alcohol Tracker" or "Alcofy - Alcohol Tracker and BAC Drink Calculator" or "Alcohol Diary" or "Alcohol Drink Calendar" or "Alcohol meter" or DrinkCoach or "DrinkControl: Alcohol Tracker" or DrinksMeter or "Dry Days by AlcoChange" or "Know Your Numbers" or "KNOW YOUR UNITS" or MeSelfControl or ReduceYourDrinking or ("Saying When" and CAMH) or "Simple Alcohol Unit Tracker" or "TRY DRY: The app for Dry January and beyond" or "Wise Drinking: Let's be smart" or "drinkcoach.org.uk" or "alcohol.org" or "HSE.ie" or "drinkaware.ie" or "CheckAlc.com").mp. |
| 3: Quantification as tool objective | (Screen* or Quantif* or Measur* or Assess* or Test* or Evaluat*).ti,ab  OR exp *Self Report/ |
| 4: Type of study | "Reproducibility of Results"/  OR exp *data accuracy/  OR exp *"sensitivity and specificity"/  OR exp *validation study/  OR exp *software validation/  OR exp Comparative Study/  OR (Comparative Study).ti,ab  OR (randomized trial OR randomized controlled trial OR randomized clinical trial).ti,ab |
