# Supplementary material for: Digital Assessment Tools Using Animation Features to Quantify Alcohol Consumption: Systematic App Store and Literature Review
Source: J Med Internet Res. 2022 Mar 23;24(3):e28927. doi: 10.2196/28927 (PMC8987963; doi:10.2196/28927)
Supplement: Multimedia Appendix 2 [file jmir_v24i3e28927_app2.pdf]

Appendix 2a – Mobile digital assessment tools quantifying alcohol consumption (alcohol DATs) in English language: Characteristics of the included tools (n= 19).

**Main feature**	**Name** (year of last update[a], country)	**Icon**	**Publishing institution (type)**	**Operating system**	**Times downloaded** from Google Play	**Target group**	**Mentions scientific background**	**Choose drinks** from visual selection	**Choose vessels** from visual selection	**Virtually pour drinks**	**Adjust drink characteristics** (non-visually)	**User feedback**		**Extra features**
												**Unit of consumption[b]**	**Additional feedback**	
**One-time assessment of risky drinking**	**Drinks Meter** (2020, United Kingdom)[55]		Global Drug Survey - GDS (private company)	Both	10 000+	A	N	Y	Y	N	Y	SD	Physiology/ nutrition (calories)	Text-based AUDIT; 'drink pourer' tool
	**Know Your Numbers** (2017, United Kingdom)[52]		Wessex Academic Health Science Network (public actor)	Both	500+	Health care profes sionals	Y	Y	Y	N	N	SD		Alcohol unit guide
	**Know Your Units** (2017, United Kingdom)[54]		Alcohol And You Northern Ireland (public actor)	Both	500+	A	N	Y	N	N	Y	SD	Physiology/ nutrition (calories)	Beverage-specific sound animations
**Individualized program to reduce or quit drinking**	**MeSelfControl** (2016, Germany)[60]		appPeople (no information)	Both	1000+	A	Y	Y	Y	N	Y	Alcohol quantity		
	**ReduceYour Drinking** (2015, Denmark)[47]		H. Lundbeck A/S (private company - pharmaceuticals)	iOS	n/a	A	N	Y	N	N	N	Alcohol quantity		Text-based DAT<br><br>Available in Russian
	**Saying When** (2016, Canada)[59]		CAMH/Centre for Addiction and Mental Health (public actor)	Both	5000+	A	Y	Y	Y	Y	N	SD	Positive effect (money saved; sober days)	Explanation of SD concept
**BAC calculator**	**alcCalc** (2014, Japan)[51]		NOHT CO., LTD. (private company)	iOS	n/a	A	N	Y	N	N	N	Alcohol quantity	Physiology/nutrition (calories; time until sober)	
	**Alcohol Diary** (2019, not provided)[63]		James Porter (no information)	iOS	n/a	A	N	Y	N	N	N	SD		
	**Alcohol meter** (2019, not provided)[61]		J. Alcaraz (no information)	Android	5000+	A	N	N	N	Y	Y	Alcohol quantity	Physiology/nutrition (time until sober)	
	**DrinkWatch Unit Checker** (2016, United Kingdom)[65]		Henry McCrory (registered charity UK)	iOS	n/a	A	N	Y			Y	SD	Physiology/nutrition (calories; time until sober)<br>negative effect (money spent)	

Appendix 2a – Mobile digital assessment tools quantifying alcohol consumption (alcohol DATs) in English language: Characteristics of the included tools (n= 19). (continued)

Main feature	Name (year of last update[a], country)	Icon	Publishing institution (type)	Operating system	Times downloaded from Google Play	Target group	Mentions scientific background	Choose drinks from visual selection	Choose vessels from visual selection	Virtually pour drinks	Adjust drink characteristics (non-visually)	User feedback: Unit of consumption[b]	User feedback: Additional feedback	Extra features
Drinking diary	**AlcoExpert** (2019, Russia)[48]		Grey Media (private company)	Android	1000+	A	N	Y	N	N	Y	Alcohol quantity	Physiology/nutrition (equivalent in volume of beer/vodka)<br><br>negative effect (heavy drinking days)	Photorealistic drink images<br><br>Available in Russian
Drinking diary	**Alcofy** (2020, not provided)[64]		JMJ Dev (no information)	Android	5000+	A	N	Y	Y	N	Y	Alcohol quantity	Physiology/nutrition (Time until sober; typical symptoms at intoxication level)	
Drinking diary	**DrinkCoach** (2020, United Kingdom)[57]		Humankind (registered charity UK)	Both	5000+	A	N	Y	N	N	Y	SD	Physiology/nutrition (calories)<br><br>positive effect (sober days)	Visualized drinking scene; link to animation-enhanced AUDIT
Drinking diary	**DrinkControl** (2020, not provided)[62]		E-protect (no information)	iOS	n/a	A	N	Y	N	N	Y	SD	Negative effect (money spent)	Photorealistic drink images
Drinking diary	**Dry Days** (2020, United Kingdom)[56]		AlcoChange (private company)	Both	10 000+	A	N	Y	Y	N	Y	SD	Positive effect (money saved; sober days)	
Drinking diary	**Drynk** (2020, Ireland)[50]		Syzible (no information)	Android	100+	A	N					SD		BAC calculator
Drinking diary	**Simple Alcohol Unit Tracker** (2020, United Kingdom)[58]		DeveloperJam (no information)	Android	10 000+	A	N	Y	N	N	N	SD	Negative effect (drinking days per week)	
Drinking diary	**TRY DRY** (2020, United Kingdom)[53]		Alcohol Change UK (registered charity UK)	Both	50 000+	A	N	Y	Y	N	Y	SD	Physiology/nutrition (calories)<br><br>positive effect (money saved; sober days)	AUDIT-C
SD counter	**Wise Drinking** (2019, France)[49]		Pernod Ricard (private company - alcoholic beverages)	Both	50 000+	A	N	Y	Y	N	Y	SD	Physiology/nutrition (calories)	Available in Russian

A adult general population. AUDIT Alcohol Use Disorder Identification Test. BAC blood alcohol concentration. DAT digital assessment tool. [g] grams. SD standard drinks.

[a] *At time of data extraction (2020)* [b] ***SD*** *= standard drinks.* ***Alcohol quantity*** *= pure ethanol consumed (e.g. in grams or liters).*

Appendix 2b – Web-based digital assessment tools quantifying alcohol consumption (alcohol DATs) in English language: Characteristics of the included tools (n= 3).

Main feature	Tool name (year of last update[a])	URL	Responsible organization (country)	Target group	Choose drinks from visual	Choose vessels from visual	Virtually pour drinks	Adjust drink characteristics	User feedback		Comments
									Unit of consumption[e]	Additional feedback[f]	
SD counter	**Drinkaware Drinks Calculator** (2020)[68]	https://www.drinkaware.ie/tools-resources/drinks-calculator	Alcohol Awareness Foundation Ireland T/A Drinkaware (Ireland)	A	Y	N	N	N	SD	Physiology/nutrition (calories; ingested sugar; hours to process)<br><br>negative effect (total cost)	Drink selection depends on chosen drinking context (home/pub)
One-time assessment of risky drinking	**DrinkCoach Alcohol Test** (not provided)[69]	https://drinkcoach.org.uk/alcohol-test	Humankind - registered charity (UK)	A	Y	Y	N	N	SD<br><br>AUDIT risk score	Physiology/nutrition (calories)	Visually enhanced AUDIT; linked to the DrinkCoach mobile tool
	**HSE Self assessment tool** (2019) [67]	https://www2.hse.ie/wellbeing/alcohol/self-assessment-tool/#Start	Health Service Executive – HSE (Ireland)	A	Y	N	N	N	SD<br><br>AUDIT risk score	Physiology/nutrition (calories; 'burger equivalent'; exercise time to burn calories)	Visually enhanced AUDIT

A adult general population. AUDIT Alcohol Use Disorder Identification Test. BAC blood alcohol concentration. DAT digital assessment tool. [g] grams. SD standard drinks.

[a]*At time of data extraction (2020)* [b]***SD*** *= standard drinks.* ***Alcohol quantity*** *= pure ethanol consumed (e.g. in grams or liters).* ***AUDIT risk score.***
